# A Laboratory Study of the McGurk Effect in 324 Monozygotic and Dizygotic Twins

**DOI:** 10.3389/fnins.2019.01029

**Published:** 2019-10-04

**Authors:** Guo Feng, Bin Zhou, Wen Zhou, Michael S. Beauchamp, John F. Magnotti

**Affiliations:** ^1^CAS Key Laboratory of Behavioral Science, Institute of Psychology, CAS Center for Excellence in Brain Science and Intelligence Technology, Chinese Academy of Sciences, Beijing, China; ^2^Department of Psychology, University of Chinese Academy of Sciences, Beijing, China; ^3^Psychological Research and Counseling Center, Southwest Jiaotong University, Chengdu, China; ^4^Department of Neurosurgery and Core for Advanced MRI, Baylor College of Medicine, Houston, TX, United States

**Keywords:** audiovisual fusion, multisensory integration, McGurk effect, twin studies, behavioral genetics

## Abstract

Multisensory integration of information from the talker’s voice and the talker’s mouth facilitates human speech perception. A popular assay of audiovisual integration is the McGurk effect, an illusion in which incongruent visual speech information categorically changes the percept of auditory speech. There is substantial interindividual variability in susceptibility to the McGurk effect. To better understand possible sources of this variability, we examined the McGurk effect in 324 native Mandarin speakers, consisting of 73 monozygotic (MZ) and 89 dizygotic (DZ) twin pairs. When tested with 9 different McGurk stimuli, some participants never perceived the illusion and others always perceived it. Within participants, perception was similar across time (*r* = 0.55 at a 2-year retest in 150 participants) suggesting that McGurk susceptibility reflects a stable trait rather than short-term perceptual fluctuations. To examine the effects of shared genetics and prenatal environment, we compared McGurk susceptibility between MZ and DZ twins. Both twin types had significantly greater correlation than unrelated pairs (*r* = 0.28 for MZ twins and *r* = 0.21 for DZ twins) suggesting that the genes and environmental factors shared by twins contribute to individual differences in multisensory speech perception. Conversely, the existence of substantial differences within twin pairs (even MZ co-twins) and the overall low percentage of explained variance (5.5%) argues against a deterministic view of individual differences in multisensory integration.

## Introduction

Humans have the remarkable ability to combine information from the mouth of a conversation partner with information from their voice in order to facilitate communication (Campbell, 2008). The interaction of visual and auditory speech can be studied with stimuli containing mismatched visual and auditory components. When presented with certain combinations of incongruent speech, such as auditory *ba* paired with visual *ga*, individuals may perceive an illusory fused percept (*da*). This illusion, first described by McGurk and MacDonald (1976), has come to be known as the McGurk effect. The effect has been widely used to examine audiovisual speech processing across the lifespan and in clinical populations (Ross et al., 1998; de Gelder et al., 2003; Burnham and Dodd, 2004; Rogers and Ozonoff, 2005; Smith and Bennetto, 2007; Tremblay et al., 2007; Pearl et al., 2009; Nath et al., 2011; Guiraud et al., 2012).

One surprising fact about the McGurk effect is that even though humans are experts in audiovisual speech perception, the illusion is not universal: even among healthy young adults, some participants never perceive the illusion, while others always do (Gentilucci and Cattaneo, 2005; Nath and Beauchamp, 2012; Stevenson et al., 2012; Strand et al., 2014; Basu Mallick et al., 2015; Magnotti and Beauchamp, 2015; Shahin, 2019). The origin of this individual variability is unknown. One obvious question is whether inherited differences in perceptual abilities influence McGurk perception, a question that can be explored with twin studies. Monozygotic twins share identical genetic makeup as well as their prenatal environment (since most MZ twins share the same placenta) while dizygotic twins share only 50% of their genetic material and never share a placenta. If MZ twins are more similar than DZ twins for a particular trait, it suggests that genetics and prenatal environment contribute to the development of the trait. The literature is mixed on the heritability of different cognitive and perceptual abilities. High heritability has been reported for some basic perceptual phenomena including binocular rivalry rate (Miller et al., 2010) and musical pitch recognition (Drayna et al., 2001); weaker heritability has been reported for olfactory sensitivity (Hubert et al., 1980), face perception (Zhu et al., 2010), and language skills such as vocabulary, syntax, and semantics (Hayiou-Thomas et al., 2012).

In order to determine the influence of genes and early environment on the McGurk effect, we examined perception of the McGurk effect in 324 healthy twins (mean age ± SD = 16.8 ± 2.1 years). 73 pairs of twins were monozygotic (MZ) and 89 pairs were same-gender dizygotic (DZ). To assess the stability of the McGurk effect, some of the participants (*n* = 150) were retested 2 years after the original test.

## Materials and Methods

### Participants

All subjects gave written informed consent to participate in an experimental protocol which was carried out in accordance with relevant guidelines and regulations and was approved by the Institutional Review Board at Institute of Psychology, Chinese Academy of Sciences. Parental informed consent was obtained for subjects under 18 years of age. The subject pool consisted of twins enrolled in the BeTwiSt twin study (Chen et al., 2013). We tested all twins who were available for testing and who met our inclusion criteria, i.e., native Mandarin speakers with normal or corrected-to-normal vision and no history of speech, language, or hearing difficulties, resulting in a sample size of 162 pairs of twins (49% male; mean age ± SD = 16.8 ± 2.1 years) comparable to that of other twin studies (Zhu et al., 2010). Seventy three pairs were monozygotic and 89 pairs were same-gender dizygotic as determined by DNA analysis of short tandem-repeat polymorphisms crosschecked with phenotypic information (Chen et al., 2010, 2013). One hundred and fifty participants underwent an additional testing session approximately 2 years after the initial session.

### Stimuli and Procedure

Stimulus presentation and response collection were controlled by a PC running E-Prime 2.0 software (Psychology Software Tools, Pittsburgh, PA, United States). The McGurk stimuli consisted of nine digital audiovisual recordings, each ∼2 s long (Basu Mallick et al., 2015; Magnotti and Beauchamp, 2015; Magnotti et al., 2015). Each stimulus was presented eight times in random order (Six subjects received 10 presentations of each stimulus; we analyze just the first 8 presentations for consistency with the rest of the subjects). Each stimulus contained an auditory recording of a syllable and a digital video of the face of the speaker enunciating a different, incongruent syllable (Table 1). There were five male speakers and three female speakers (the same female speaker appeared in two stimuli). Subjects were instructed to pay attention to each movie clip and report their percept by typing it via a standard keyboard into a computer. An open-choice design was used to minimize demand characteristics; no feedback was given. The auditory volume (∼75 dB) and the visual angle (∼5.2 × 6.0°) of the stimulus were fixed across subjects. Co-twins were tested separately, but always on the same day and in the same testing room, so that variability in testing environment, if any, was comparable for MZ pairs and DZ pairs. In order to minimize testing time, there was no auditory-only or congruent audiovisual condition: an earlier study (Basu Mallick et al., 2015) using the same McGurk stimuli showed near-perfect accuracy for identifying auditory-only and congruent audiovisual syllables (accuracy of 98 and 99%, respectively).

**TABLE 1:** McGurk stimuli.

**Stimulus#**	**S1**	**S2**	**S3**	**S4**	**S5**	**S6**	**S7**	**S8**	**S9**
Auditory component	Ba	Ba	Baba	Baba	Baba	Ba	Ba	Pa	Pa
Visual component	Ga	Ga	Gaga	Gaga	Gaga	Ga	Ga	Ka	Ka
McGurk percept	Da	Da	Dada	Dada	Dada	Da	Da	Ta	Ta

*The stimuli consisted of 9 movie clips showing 8 talkers (5 males, 3 females; 1 female appeared in two movie clips) speaking syllables in which the voice (auditory component) and mouth movements (visual component) were incongruent.*

#### Analyses

Subject responses were classified into four categories: auditory (responses corresponding to the auditory syllables), visual (responses corresponding to the visual syllables), McGurk (specific responses containing an element not contained in either the auditory or visual syllable, described in the original paper as “fused” responses, Table 1), and other (responses different from the previous three categories, e.g., “a”). For stimuli with double syllables (S3, S4, S5 in Table 1) each syllable in the response was coded independently (e.g., the response “dada” was coded as 1.0 McGurk; the response “daba” was coded as 0.5 McGurk and 0.5 auditory). The mean proportion of McGurk percepts across all presentations of all movie clips indexed one’s susceptibility to the McGurk effect.

To ensure our stimuli elicited a reliable McGurk effect in native Mandarin speakers, we compared a subset of the participants in the current study (the older twin of each twin pair) with a sample of native English speakers from the United States (Magnotti et al., 2015). Using the same stimuli and a similar open-choice procedure, we found no statistical difference between mean McGurk susceptibility for the Mandarin speakers (0.48) and the English speakers (0.44). Individuals from both groups spanned the entire range of McGurk perception (from 0 to 1) demonstrating a similar degree of interindividual variability across cultures.

To assess the consistency of individual differences, we tested both within-test reliability (odd-even split-half reliability) and test-retest reliability (Pearson’s correlation) for the proportions of McGurk responses. For assessing the statistical significance of individual Pearson correlations, we report the associated 95% confidence interval and the results of the *t*-test assessing the null hypothesis of no linear relationship between the two variables being correlated.

To assess the similarity of McGurk illusion perception between cotwins, we separately calculated intraclass correlations for MZ and DZ pairs using the packge “irr” in R and report the corresponding analysis of variance (ANOVA). To compare these correlations, we used Fisher’s r-to-z transform to test the null hypothesis that the correlations are equal.

As a secondary measure of similarity, we determined how accurately we could predict each subject’s McGurk illusion perception (root mean squared error across stimuli) using the co-twin’s illusion perception. As a comparison, we also used the mean value across all subjects (except the subject being predicted) to predict each subject’s illusion perception. To compare error rates between these methods, we used a non-parametric Wilcoxon signed–rank test.

We conducted additional control analyses to assess the robustness of our presented results. First, we used bootstrapped simulations to calculate the expected intraclass correlation between unrelated pairs of individuals for both illusion percepts and auditory percepts (visual and “other” responses were of lower frequency and were not examined). Next we used a linear mixed-effect model to assess the relationship between illusion perception and the collected demographic variables (fixed factors of age, gender, birth order, and all their interactions), with random effects of subject and stimulus. Finally, we looked for mean differences in the percentages of response types (auditory, illusion, visual, or other) between MZ and DZ twins.

## Results

**FIGURE 1:**
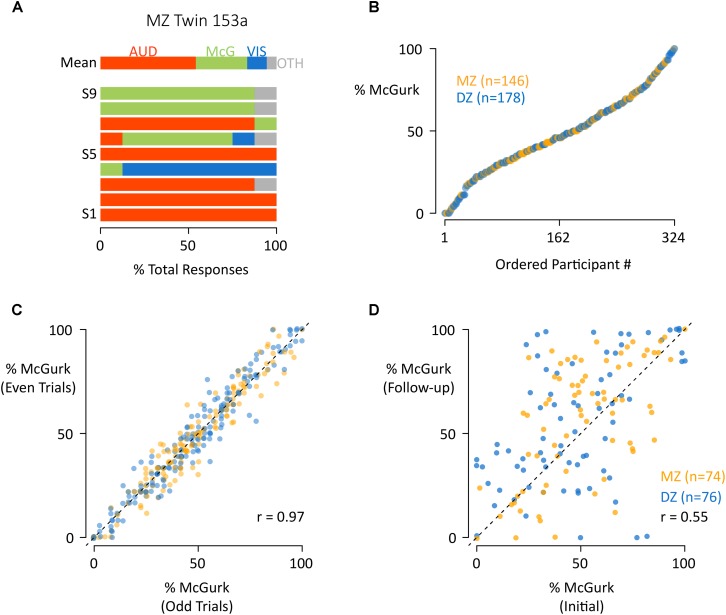
The distribution and reliability of susceptibility to the McGurk effect. **(A)** For a single participant (MZ Twin 153a) the mean response across stimuli (row “Mean”) and for each individual stimulus (successive rows) are shown. For each stimulus presentation, the participant reported a percept, which was classified as auditory (AUD, red), McGurk (McG, green), visual (VIS, blue), or other (OTH, gray). The colored portions of each bar illustrate the percentage of each response type across repeated presentations of the same stimulus. For instance, the red color of the bar labeled “S1” indicates that the participant reported an auditory percept for every presentation of stimulus “S1.” **(B)** For each participant, the mean percentage of McGurk reports across all stimuli (“% McGurk”) was calculated across stimuli, equivalent to the width of the green bar in the first row of **(A)**. Participants were ordered by % McGurk and plotted, one symbol per participant, orange for MZ twins, blue for DZ. **(C)** To assess reliability, for each participant the mean percentage of McGurk reports to even and odd trials were plotted, one symbol per participant, orange for MZ twins, blue for DZ. **(D)** In a follow-up experimental session approximately 2 years after the initial testing session, participants responded to the same stimuli. To assess reliability, for each participant the mean percentage of McGurk reports at the initial and follow-up sessions were plotted, one symbol per participant, orange for MZ twins, blue for DZ.

Different McGurk stimuli vary in efficacy, so we presented nine different McGurk stimuli in order to obtain a more generalizable estimate of each participant’s susceptibility to the illusion. Seven of the stimuli consisted of pairings of auditory “ba” and visual “ga” and two of the stimuli consisted of pairings of auditory “pa” and visual “ka” (Table 1). Participants used an open-choice response with no feedback to reduce demand characteristics. Responses were classified into four categories: auditory, visual, McGurk illusory fusion, and other (see Supplementary Data Sheet S1). Consistent with previous studies, there was substantial variability across different stimuli within the same participant. For instance, for stimulus 1 in the participant shown in Figure 1A, all of the responses corresponded to the auditory component of the stimulus while for stimulus 9 in the same participant, nearly all responses corresponded to the illusory fusion percept. Across participants, the most frequent report was the illusory fusion percept (48 ± 24%, mean percentage ± SD) followed by reports of the auditory component of the stimulus (25 ± 22%). There were fewer visual (12 ± 12%) and other responses (16 ± 14%).

As suggested by the large standard deviations in the response percentages, there was substantial variability across participants. We defined the McGurk susceptibility for each subject as the mean proportion of McGurk fusion reports across all presentations of all stimuli. A plot of the susceptibility values showed a broad range, with some subjects never reporting the effect and others always reporting it (Figure 1B). This broad distribution could not be explained by random response selection, as subjects responded consistently: across even and odd trials within the first testing session, the Pearson correlation for the proportion of McGurk responses was 0.97 [95% CI: 0.96 to 0.97, 95% CI; *t*(322) = 68.1, *p* < 10^–16^; Figure 1C]. Across the first and second testing session, separated by 2 years (see Supplementary Data Sheet S2), the Pearson correlation was 0.55 [95% CI: 0.42 to 0.65; *t*(148) = 7.9, *p* = 10^–13^; Figure 1D].

**FIGURE 2:**
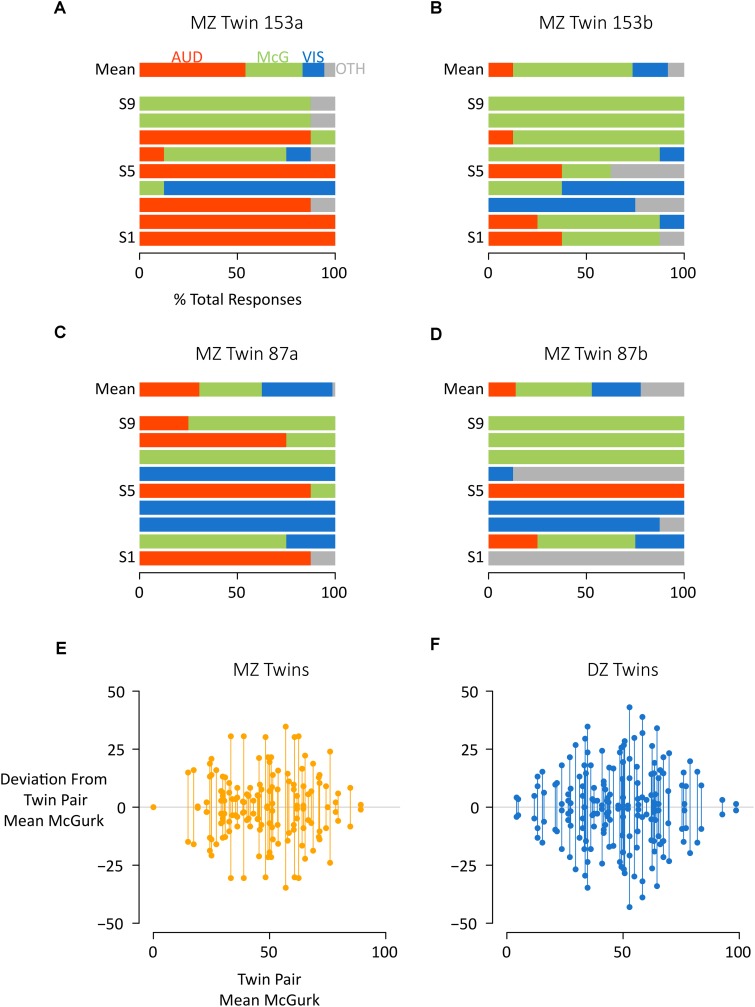
Comparing McGurk susceptibility between and across co-twins. **(A)** For a single participant (MZ Twin 153a) the mean response across stimuli (row “Mean”) and for each individual stimulus (successive rows) are shown. For each stimulus presentation, the participant reported a percept, which was classified as auditory (AUD, red), McGurk (McG, green), visual (VIS, blue), or other (OTH, gray). The colored portions of each bar illustrate the percentage of each response type across repeated presentations of the same stimulus. For instance, the red color of the bar labeled “S1” indicates that the participant reported an auditory percept for every presentation of stimulus “S1.” **(B)** For the other co-twin in this pair (MZ Twin 153b) a different pattern of responses was noted. For instance, for stimulus “S1”, a majority of participant reports corresponded to the McGurk percept. **(C,D)** Co-twins MZ Twin 87A and 87B showed more similar responses across stimuli. **(E,F)** To examine individual twin pairs, the % McGurk reports within each twin pair was calculated, and the deviation from this mean plotted by showing two circles (one for each co-twin) connected by a vertical line. The average length of the vertical lines indicates the magnitude of the intraclass correlation. The left plot shows all MZ twins (orange circles), the right plot shows all DZ twins (blue circles). Within each plot, twin pairs were ordered by mean% McGurks, from least to most. For instance, the left-most orange symbol pair shows two co-twins both with 0% McGurk, while the right-most blue symbol shows two co-twins with 97 and 100% McGurk.

Given that individual differences in McGurk susceptibility were reliable, we next examined the effect of twin status. For some pairs of twins, there were substantial differences between twins (such as MZ twin pair #153, Figures 2A,B). For other twin pairs, responses were more similar between co-twins (MZ twin pair #87, Figures 2C,D).

To visualize these relationships, we calculated the mean susceptibility to the McGurk effect of each twin pair and plotted the distance from the mean for each co-twin for both MZ (Figure 2E) and DZ twins (Figure 2F). For quantification, we calculated intraclass correlations (Griffin and Gonzalez, 1995) for each twin type. The mean rate of McGurk percepts was significantly correlated for both MZ twins (*r* = 0.28, 95% CI: 0.06 to 0.48, *F*(72,73) = 1.79, *p* = 0.007) and DZ twins (*r* = 0.21, 95% CI: −0.01 to 0.40 *p* = 0.026). The intraclass correlation was not significantly different between MZ and DZ twins (*z* = 0.52, *p* = 0.60). The rate of auditory percepts also showed a positive intraclass correlation for both MZ twins [*r* = 0.25, 95% CI: 0.02 to 0.45, *F*(72,73) = 1.65, *p* = 0.02] and DZ twins [*r* = 0.14, 95% CI: −0.07 to 0.34, *F*(88,89) = 1.32, *p* = 0.10] with no significant difference between MZ and DZ twins (*z* = 0.7, *p* = 0.48).

While the twin correlations were significantly different than zero, they were low in absolute terms. An *r* = 0.23 (intraclass correlation calculated across MZ and DZ twins) corresponds to only 5.5% of variance explained. As another way to understand this relatively low correlation, we estimated how well one twin pair’s response to a particular stimulus could predict their co-twin’s response to the same stimulus. Across subjects, prediction error (root-mean squared error) was 44 ± 17% for a given stimulus. Strikingly, if we instead use the mean value for a given stimulus across all subjects (except the subject being predicted), prediction error improved to 35 ± 10% for a given stimulus (*p* < 10^–16^, Wilcoxon signed–rank test).

### Control Analyses

First, we verified that the intraclass correlations between actual twins (MZ and DZ twins combined, *r* = 0.25 and 0.17 for McGurk and auditory percepts, respectively) were higher than would be expected for randomly selected non-twin pairs for both McGurk illusion percepts (mean simulated |*r*| < 0.01, 95% bootstrap interval CI: −0.14 to 0.13) and auditory percepts (mean |*r*| < 0.01, 95% CI: −0.14 to 0.12).

Second, we examined demographic explanations for individual differences in McGurk susceptibility. Age, gender, birth order, and their interactions failed to explain a significant portion of the variance in McGurk susceptibility (linear mixed effects model with gender, age, birth order, and all two- and three-way interactions as fixed factors; subject and stimulus were random effects; all parameter tests yielded |*t*| < 1.15, *p*s > 0.25).

Finally, we verified that there were no mean differences between MZ and DZ groups in any percept category: McGurk, MZ vs. DZ, 48 ± 23% vs. 48 ± 25%, *t*(322) = −0.06, *p* = 0.95; auditory: 23 ± 22% vs. 26 ± 22%, *t*(322) = 0.96, *p* = 0.34; visual: 13 ± 13% vs. 12 ± 11%, *t*(322) = −0.97, *p* = 0.33; other: 16 ± 15% vs. 15 ± 13%, *t*(322) = −0.56, *p* = 0.58.

## Discussion

Twin studies have a long and troubled history of being used to support unjustified conclusions that human behavior is strongly genetically determined, reviewed in Joseph (2015). Strong claims about the heritability of behavior rest on many assumptions, including the conjecture that MZ and DZ twins experience equal environments, so that greater behavioral similarity between MZ than DZ co-twins can only be explained by greater genetic similarity. However, *in utero*, approximately two-thirds of MZ twins share placentas, while no DZ twins share placentas. Since most MZ co-twins are exposed to exactly the same concentration and temporal variation in developmentally active biochemical factors in their shared circulation, MZ twins have much more similar prenatal environments than DZ twins, invalidating the equal environment assumption.

Keeping these caveats in mind, we found that the degree of susceptibility to the McGurk effect in MZ and DZ twins was significantly more similar than chance, demonstrating that shared genes and environment play some role in determining McGurk susceptibility. However, the percent variance explained (*r*^2^) was only 5.5% (averaged across MZ and DZ twins) and there was no significant difference between MZ and DZ twins. Put another way, for both MZ and DZ twins, knowing how often one co-twin perceived the McGurk effect provided little information on the McGurk perception of the other co-twin.

This result argues against a deterministic view of individual differences in the McGurk effect. A growing body of literature shows that McGurk susceptibility is significantly, but weakly, correlated with a number of cognitive and personality factors, including the pattern of eye movements made when viewing a talking face (Gurler et al., 2015); lipreading skill (Strand et al., 2014; Brown et al., 2018); temporal binding window (Stevenson et al., 2012); and autistic traits (Van Laarhoven et al., 2019). Taken together, these findings suggest that audiovisual speech perception, as assayed with the McGurk effect, is similar to other complex human behaviors in that it is influenced by a host of factors, both genetic and environmental.

### Stimulus Variability

The audiovisual recordings of *ba*/*ga* and *pa*/*ka* used by McGurk and MacDonald in their original study are lost (McGurk and MacDonald, 1976; Beauchamp, 2018; MacDonald, 2018) forcing each laboratory to create their own stimuli. These stimuli vary in efficacy, hindering across-study comparisons (Alsius et al., 2018). Other studies have reported high interstimulus variability across different stimulus exemplars of *ba*/*ga* (Jiang and Bernstein, 2011; Basu Mallick et al., 2015), *pa*/*ka* (Magnotti and Beauchamp, 2015) and *ba*/*fa* (Shahin et al., 2018; Shahin, 2019).

Many factors, including co-articulation (number of repetitions of the syllable, i.e., *ba* vs. *baba*) and speech rate (fast vs. slow) may contribute to the efficacy of a given McGurk exemplar (Magnotti et al., 2018a, b). In the absence of techniques for psychometrically manipulating McGurk stimuli as there are for simpler sensory stimuli (such as parametrically changing the volume of a tone to measure auditory sensitivity) participants in the present study viewed a battery of nine different *ba/ga* and *pa/ka* McGurk exemplars, with the average score across stimuli measuring the overall susceptibility of a given participant to the illusion. Because the same stimulus battery was presented to every individual, comparing scores within and across twin pairs allowed for an estimate of the magnitude of genetic and early environmental influences on McGurk susceptibility. Adding additional stimuli to the battery, such as *ba*/*fa* (Shahin, 2019), would increase generalizability at the cost of additional experimental time.

### Participant Variability

In addition to interstimulus variability, our study confirms and extends previous reports demonstrating that different individuals vary greatly in their susceptibility to the McGurk effect (Gentilucci and Cattaneo, 2005; Nath and Beauchamp, 2012; Strand et al., 2014; Basu Mallick et al., 2015; Magnotti and Beauchamp, 2015; Shahin, 2019). Our study used a sample size (*n* = 324) that is an order of magnitude larger than most published studies of the McGurk effect. This is important because in a phenomenon with high inter-subject variability, large sample sizes (>100) are necessary to accurately estimate differences between groups (Magnotti and Beauchamp, 2018).

In order to obtain large sample sizes, online testing services such as Amazon Mechanical Turk make it possible to quickly and easily present stimuli to many participants. Although we have previously shown similar distributions of McGurk illusion perception between online and laboratory settings (Experiment 4 in Magnotti et al., 2018b), a concern with on-line testing of audiovisual speech is that different participants are likely to view the stimuli in different formats (e.g., a small tablet screen vs. a large monitor) and hear them under different listening conditions (e.g., headphones vs. loudspeakers; quiet vs. noisy environments) that are not under the control of the investigator. The present study used in-person testing in the laboratory, rather than on-line testing, allowing viewing and listening conditions to be carefully controlled and equated across participants. Therefore, the observed inter-subject variability cannot be attributed to the differences in viewing and listening conditions that are a possible confound in on-line testing. Given that there was only a small main effect of heritability/early environment on McGurk perception, we did not investigate the interaction between heritability/early environment and interstimulus or interparticipant variability.

### Cultural Variability

All study participants were native speakers of Mandarin, while the McGurk stimuli were recorded by English speakers. However, the English syllables contained in the stimuli are common sounds in Mandarin, serving as acoustic subunits of Mandarin words with identical spellings in Pinyin, the official Romanization system for Standard Chinese. Although the McGurk effect was first discovered in native English speakers, previous studies have demonstrated that it is a cross-cultural phenomenon experienced by Mandarin speakers as well as native speakers of Cantonese, Finnish, German, Hebrew, Hungarian, Italian, Japanese, Spanish and Thai (Sekiyama and Tohkura, 1991; Grassegger, 1995; Aloufy et al., 1996; Fuster-Duran, 1996; Sekiyama, 1997; Burnham and Lau, 1998; Sams et al., 1998; Chen and Hazan, 2007; Traunmüller and Öhrström, 2007; Bovo et al., 2009).

In order to examine McGurk differences between cultures, in a previous publication we compared a subset of the participants in the current study (the older twin of each twin pair, *n* = 162) with a sample of native English speakers from the United States (*n* = 145) (Magnotti et al., 2015) using the same stimuli and a similar open-choice experimental procedure, we found no significant difference in mean McGurk susceptibility between native Mandarin and native English speakers (48 ± 2% vs. 44 ± 2%) and individuals from both groups spanned the entire range of McGurk susceptibility (from 0 to 100%). In the absence of strong intercultural differences in the illusion, there is no reason to think that the relatively small effect of genes and environment on McGurk perception estimated for native Chinese speakers would be larger for native speakers of other languages, including English.

## Data Availability Statement

The datasets analyzed during the current study are included as Supplementary Material to this manuscript.

## Ethics Statement

This study involving human participants were reviewed and approved by all subjects gave written informed consent to participate in an experimental protocol which was carried out in accordance with relevant guidelines and regulations and was approved by the Institutional Review Board at Institute of Psychology, Chinese Academy of Sciences. Parental informed consent was obtained for subjects under 18 years of age. Written informed consent to participate in this study was provided by the participants’ legal guardian/next of kin.

## Author Contributions

MB conceived the study. MB and WZ designed the study. GF and BZ performed the testing and data collection. GF and JM performed the data analysis and interpretation under the supervision of WZ and MB. All authors contributed to the writing of the manuscript and approved the final version of the manuscript for submission.

## Conflict of Interest

The authors declare that the research was conducted in the absence of any commercial or financial relationships that could be construed as a potential conflict of interest.

## Supplementary Material

DATA SHEET S1 Participants’ responses to McGurk stimuli presented in the initial study of Experiment 1.

DATA SHEET S2 Participants’ responses to McGurk stimuli presented during follow-up study.
